# Challenges and Parental Perspectives on Sexual Education for Children and Adolescents in Tunisia: A Cultural Insight

**DOI:** 10.1192/j.eurpsy.2025.451

**Published:** 2025-08-26

**Authors:** A. Hadj Salah, S. Iben Khedher, A. Haouala, I. Batbout, M. Ben Mbarek, F. Zaafrane, A. Mhalla, B. Amamou

**Affiliations:** 1 Psychiatry, Fattouma Bourguiba Hospital, Monastir, Tunisia

## Abstract

**Introduction:**

School-based sexual education aims to provide children and adolescents with the knowledge and values necessary for a positive view of their sexuality within their emotional and social development. However, sexual health education in Tunisian schools remains controversial, particularly among parents.

**Objectives:**

To assess parents’ perceptions of school-based sexual education and identify challenges related to sexual health education in Tunisia.

**Methods:**

This descriptive study, conducted from September to October 2021, involved 154 parents from various Tunisian governorates. Participants completed a self-administered questionnaire in classical Arabic distributed via online groups. The survey explored perceptions of sexual education and identified key challenges.

**Results:**

Of the participants, 55.8% (n=86) were aware of the national sexual education program, but only 13.6% (n=21) knew its content. A majority (81.8%; n=126) supported school-based sexual education. Key topics for discussion included the human body and its development (77.3%), moral values, rights, and culture (68.8%), and violence and safety (68.2%). Most parents preferred discussions to start in middle school (46.1%; n=71) or primary school (30.5%; n=47), with only 6.5% (n=10) advocating for earlier education.

Parents, healthcare professionals, and teachers were seen as primary educators (68.2%; n=105; 64.3%; n=99; 63%; n=97). While 55.8% recognized sexual rights as part of human rights, only 39.53% (n=34) were knowledgeable about these rights. Only 29.1% of parents (n=46) regularly discussed sexuality with their children. Despite the general value placed on sexual education and minimized concerns about negative consequences, some parents worried about risks such as early sexual activity. On a scale of 1 to 10, half of the participants (50%; n=77) rated the importance of sexual education at 8/10 or higher.

**Conclusions:**

The study highlights the need for sexual education programs tailored to Tunisia’s cultural context. Despite strong parental support (81.8%) and acknowledgment of essential topics like human development and moral values, only 13.6% are familiar with the program content. Challenges include concerns about cultural fit and potential negative impacts. Engaging parents, educators, and health professionals is crucial for overcoming these challenges. Further research should explore these cultural dynamics and assess.

**Disclosure of Interest:**

None Declared

